# Cerebrospinal fluid catecholamines in Alzheimer’s disease patients with and without biological disease

**DOI:** 10.1038/s41398-022-01901-5

**Published:** 2022-04-09

**Authors:** Kristi Henjum, Leiv Otto Watne, Kristin Godang, Nathalie Bodd Halaas, Rannveig Saksholm Eldholm, Kaj Blennow, Henrik Zetterberg, Ingvild Saltvedt, Jens Bollerslev, Anne Brita Knapskog

**Affiliations:** 1 Department of Geriatric Medicine, Institute of Clinical Medicine, Oslo University Hospital, Ullevaal, University of Oslo, Oslo, Norway; 2 Department of Geriatric Medicine, Oslo University Hospital, Ullevaal, Oslo, Oslo, Nydalen, Norway; 3 Section of Specialized Endocrinology, Department of Endocrinology, Oslo University Hospital, Oslo, Norway; 4 Center for Lifespan Changes in Brain and Cognition, Department of Psychology, University of Oslo, Oslo, Norway; 5 Department of Neuromedicine and Movement Science, Norwegian University of Science and Technology, Trondheim, Norway; 6 Department of Geriatrics, St. Olavs Hospital, University Hospital of Trondheim, Trondheim, Norway; 7 Department of Psychiatry and Neurochemistry, Institute of Neuroscience and Physiology, The Sahlgrenska Academy at the University of Gothenburg, Mölndal, Sweden; 8 Clinical Neurochemistry Laboratory, Sahlgrenska University Hospital, Mölndal, Sweden; 9 Department of Neurodegenerative Disease, UCL Institute of Neurology, Queen Square, London, UK; 10 UK Dementia Research Institute at UCL, London, UK; 11 Faculty of Medicine, University of Oslo, Oslo, Norway

**Keywords:** Biomarkers, Psychology

## Abstract

Noradrenergic and dopaminergic neurons are involved in cognitive functions, relate to behavioral and psychological symptoms in dementia and are affected in Alzheimer’s disease (AD). Amyloid plaques (A), neurofibrillary tangles (T) and neurodegeneration (N) hallmarks the AD neuropathology. Today, the AT(N) pathophysiology can be assessed through biomarkers. Previous studies report cerebrospinal fluid (CSF) catecholamine concentrations in AD patients without biomarker refinement. We explored if CSF catecholamines relate to AD clinical presentation or neuropathology as reflected by CSF biomarkers. CSF catecholamines were analyzed in AD patients at the mild cognitive impairment (MCI; *n* = 54) or dementia stage (*n* = 240) and in cognitively unimpaired (*n* = 113). CSF biomarkers determined AT status and indicated synaptic damage (neurogranin). The AD patients (*n* = 294) had higher CSF noradrenaline and adrenaline concentrations, but lower dopamine concentrations compared to the cognitively unimpaired (*n* = 113). AD patients in the MCI and dementia stage of the disease had similar CSF catecholamine concentrations. In the CSF neurogranin positively associated with noradrenaline and adrenaline but not with dopamine. Adjusted regression analyses including AT status, CSF neurogranin, age, gender, and *APOEε4* status verified the findings. In restricted analyses comparing A+T+ patients to A−T− cognitively unimpaired, the findings for CSF adrenaline remained significant (*p* < 0.001) but not for CSF noradrenaline (*p* = 0.07) and CSF dopamine (*p* = 0.33). There were no differences between A+T+ and A−T− cognitively unimpaired. Thus, we find alterations in CSF catecholamines in symptomatic AD and the CSF adrenergic transmitters to increase simultaneously with synaptic damage as indexed by CSF neurogranin.

## Introduction

Alzheimer’s disease (AD) is the most common cause of dementia [1]. Along with the deposition of amyloid-beta (Aβ; A) and neurofibrillary tangles (NFTs; T), neurodegeneration (N) with brain atrophy in certain brain regions defines the AD neuropathology [2]. As targets of AD therapeutics, the cholinergic and glutamatergic neurotransmitter system have received researchers’ attention. However, dopaminergic and noradrenergic neurons are also afflicted in AD [3]. Together with adrenaline, noradrenaline and dopamine comprise the catecholamines.

Behavioral and psychological symptoms in dementia (BPSD) are associated with changes in catecholamine transmission [4]. Noradrenaline’s function relates to features of the AD clinical presentation such as cognition including memory, sleep-wake regulation, mood and stress and modulates neuroinflammation [5–9]. The primary source of brain noradrenaline is the pontine locus coeruleus (LC) nucleus [5]. In the aspect of AD, the LC is of particular interest [10, 11], as the LC is of the first regions to present with tau accumulations as pretangles and NFTs [12, 13]. Reduction in the LC neuronal numbers is evident at symptomatic presentation and progress with the disease [14–16]. The projections from LC to the hippocampus and amygdale may however be affected earlier [17]. As these alterations occur at the prodromal stages, they may be a target of future AD therapeutics. Phenylethanolamine-N-methyltransferase (PNMT) converts noradrenaline to adrenaline. Brain PNMT expression is scarce and predominantly locates to neuronal clusters in C1 and C2 in the medulla. C1 neurons project to the LC, but the signaling role of adrenaline is unsettled [18].

Dopaminergic neurons originate from the substantia nigra and the ventral tegmental area (VTA). While the substantia pars compacta (SNpc) form pathways associated with movement, the VTA is, among other things, associated with reward and cognitive functions, such as memory [3, 6]. Alterations in the dopaminergic system are also reported in AD [3, 19]. Although both the SNpc and the VTA are affected in AD [20] the SNpc is more affected in dementia with Lewy bodies (DLB) [21, 22]. In a transgenic mouse model of Aβ pathology, dopaminergic neuronal loss was confined to the VTA and preceded Aβ deposition [23]. Affection and reduced activity of the VTA as an early event in AD is corroborated in human studies [24].

Biomarkers now allow in vivo determination of the AD AT(N) status [25, 26]. Thus, an AD clinical diagnosis, including cognitive severity staging, may be refined by the AT(N) framework. In addition, people with preclinical AD, i.e., cognitively unimpaired with existing AD neuropathology, may be identified [25, 26]. The post-synaptic protein neurogranin [27] is expressed in the associative cortex, hippocampus, striatum, and amygdalae [28]. Neurogranin has emerged as a cerebrospinal fluid (CSF) biomarker of AD related synaptic damage [29–31]. Reports of CSF catecholamines and their metabolites are inconsistent in AD [32–37]. However, CSF catecholamine concentrations have mainly been studied in patients with AD clinical diagnoses without biomarker support. Thus, clinical heterogeneity might have masked an association between CSF catecholamine concentrations and AD.

We aimed to explore if CSF catecholamine concentrations relate to the AD clinical diagnosis, AD clinical stage, or AD neuropathology, including synaptic damage as reflected by CSF biomarkers. CSF catecholamines were analyzed in clinically diagnosed AD patients at the mild cognitive impairment (MCI) or dementia stage with cognitively unimpaired as reference. CSF measures were applied to secondarily determine AT(N) state. As cognitive impairment in AD seems related to synaptic damage [38], the association between CSF catecholamines and CSF neurogranin, as a marker of AD related synaptic damage, were explored in addition to the AT(N) markers.

## Materials/subjects and methods

### Study participants

The study-setup was cross-sectional, including memory clinic patients with a clinical presentation of AD or mixed AD and cerebrovascular disease at different disease stages (MCI and dementia) and cognitively unimpaired (Table 1).

**Table 1:** Characteristics of the whole cohort.

	Memory clinic cohort			CU	*p* values	
	All Patients	AD-MCI patients	AD dementia		All patients vs. CU	AD-MCI vs. AD Dementia
Patient characteristics	*N* = 294	*N* = 54	*N* = 240	*N* = 113		
Age	70.4 (6.4)	71.3 (5.3)	70.2 (6.7)	72.3 (6.0)	**0.007a**	0.20a
Women	171 (58.2)	33 (61.1)	138 (57.5)	54 (47.8)	0.06b	0.63b
Education	11.9 (3.6)	12.7 (3.9)	11.7 (3.5)	14.1 (3.5)	**<0.001a**	0.06a
*APOE ε4*-positive^*^	190 (73.9)	34 (70.8)	156 (74.6)	42 (38.9)	**<0.001b**	0.59b
Cognition						
MMSE	23.1 (4.4)	26.3 (3.1)	22.4 (4.3)	29.2 (0.9)	**<0.001a**	**<0.001**
CDT accepted	146 (52.0)	41 (80.4)	105 (45.7)	108 (95.6)	**<0.001b**	**<0.001b**
TMT A ≥ −2 SD	171 (64.0)	43 (87.8)	128 (58.7)	106 (93.8)	**<0.001b**	**<0.001b**
TMT B ≥ −2 SD	109 (43.3)	35 (72.9)	74 (36.3)	105 (92.9)	**<0.001b**	**<0.001b**
BPSD						
NPI-Q^†^	3.4 (3.8)	2.0 (2.9)	3.7 (3.9)			**0.004a**
CSDD^††^	4.6 (4.1)	2.8 (3.6)	5.0 (4.1)			**0.001a**
CSF biomarkers						
Noradrenaline pmol/l	17,871.1 (8109.3)	18,986.2 (8985.8)	17,620.2 (7897.4)	14,998.9 (8062.7)	**0.001a**	0.37a
Adrenaline pmol/l	12,944.3 (6120.5)	13,720.1 (6123.0)	12,769.8 (6119.1)	6225.6 (3382.5)	**<0.001a**	0.16a
Dopamine pmol/l	2159.4 (898.7)	2374.7 (863.1)	2110.9 (901.2)	2515.7 (934.0)	**<0.001a**	0.18a
Neurogranin pg/ml^†††^	247.2 (86.9)	231.0 (78.2)	250.7 (88.3)	209.5 (70.7)	**<0.001a**	0.22a
Amyloid β42 pg/ml	561.2 (167.0)	604.3 (174.5)	551.5 (164.1)	705.2 (206.8)	**	**0.04a**
Total tau pg/ml	704.9 (368.0)	561.2 (271.2)	737.3 (379.5)	368.7 (149.2)	**	**0.001a**
Phoshorylated tau_181_ pg/ml	88.5 (37.3)	76.5 (30.5)	91.2 (38.1)	59.5 (20.1)	**	**0.008a**

Bold values identify statistical significance.

Data are presented as *N* (%) and mean (SD)

*MCI* mild cognitive impairment, *AD* Alzheimer’s disease, *CU* cognitively unimpaired, *MMSE* mini mental state examination, *CDT* clock drawing test, *TMT* trail making test, *NPI-Q* neuropsychiatric inventory questionnaire, *CSDD* Cornell scale of depression in dementia.

a = *T*-test.

b = Chi square.

^%^Valid percent without missing.

*Missing genotype data in *n* = 42, **Comparison not possible due to inter-laboratory variability.

^†^Missing in 13 patients.

^††^Missing in 49 patients.

^†††^Missing in 91 patients and one CU.

### Memory clinic patients

The study included patients admitted to the memory clinics at Oslo University Hospital (OUH, *n* = 176) and St. Olav University Hospital (*n* = 143), with a clinical presentation of probable or possible AD or AD mixed with cerebrovascular disease according to the core clinical NIA-AA 2011 criteria [39, 40]. Mixed presentations among the patients due to other causes (for instance, depression and other neurodegenerative disorders) were excluded. Experienced physicians assessed the patients according to a standardized research protocol utilizing information from the patients, their caregivers, and general practitioners [41]. The patients underwent a physical examination and cognitive tests. Computer tomography (CT) or magnetic resonance imaging (MRI) brain scans were also obtained. Cognitive tests included among others the Mini-Mental State Examination (MMSE), the Consortium to Establish a Registry of Alzheimer’s Disease (CERAD), 10-item word list and figure copying, the Clock Drawing Test (CDT), and the Trail Making Tests A and B (TMT A and B). Behavioral and psychological symptoms were assessed with the Neuropsychiatric Inventory Questionnaire (NPI-Q). The severity sum score of the 12 neuropsychiatric symptoms was calculated.

The patients underwent a diagnostic lumbar puncture, all before 11 AM, CSF for research purposes was obtained in cryotubes after the routine samples were collected. The cryotubes were centrifuged at 2000×*g* for 10 min, allocated into smaller cryotubes and immediately frozen at −20 °C. Within 1 week, the samples were moved to −80 °C. The AD core biomarkers amyloid-beta 1–42 (Aβ42), phosphorylated-tau_181_ (P-tau), and total-tau (T-tau), in CSF were analyzed at Akershus University Hospital (AHUS) by enzyme-linked immunosorbent assays (ELISA; Innotest® hTau Ag, phoshoTau (181P) and β-amyloid 1–42 Fujirebio Europe, Gent, Belgium) [42–44]. CSF measurements were used to determine AT(N) categories, i.e., biological AD status. Specific cut-offs provided by the laboratory were applied as follows: A+; Aβ42 < 700 pg/ml, T+; P-tau > 80 pg/ml while N+ was denoted by age-adjusted cut-off concentrations for T-tau > 300 pg/ml for patients under the age of 50, 450 pg/ml for those aged 50–70 years, and >500 pg/ml for those older than 70 years.

### Cognitively unimpaired

Patients undergoing elective surgery for gynecological, orthopedic, or urological problems were recruited as a cognitively unimpaired control group (cognitively unimpaired; *n* = 113). These underwent the same cognitive tests as the memory clinic patients and those with normal results at baseline were initially included. The majority was also tested after two years, and those with abnormal test results at follow-up were excluded. Patients were also excluded if they had sequela after stroke, Parkinson’s disease, or other neurodegenerative diseases likely affecting cognition (at baseline). CSF was obtained in conjunction with spinal anesthesia at the daytime surgery (between 08–11:00: 55 patients, 11:01–13.00: 29 patients, 13.01–14.30: 21 patients after 15.00: 3 patients). Time of sampling was missing for two patients. The first drops of CSF were discarded and CSF thereafter obtained in cryotubes until a maximum of 12 ml. Further details are previously described for this cohort [45]. CSF AD core biomarkers were analyzed at Sahlgrenska University Hospital (Mölndal, Sweden) by use of INNOTEST ELISAs as described for the memory clinic patients. For determination of AT(N) status, laboratory specific cut-offs were applied: A+; Aβ42 < 530 pg/ml, T+; P-tau181> 60 pg/ml, and N+; T-tau > 350 pg/ml [46]_._

### CSF catecholamine measurements

CSF catecholamines concentrations were determined by high performance liquid chromatography with electrochemical detection (HPLC-ECD) by use of reagents from Chromsystems (#5000). An internal standard, 3,4-dihydroxybenzylamine (DHBA) was added to all CSF samples and CSF samples run after the calibration standards. The coefficients of variation (CV) were 4.7, 6.2, and 8.3% for noradrenaline, adrenaline, and dopamine, respectively, as calculated by a CSF pooled sample injected for every tenth injection. Further details are described in a previous publication [47].

### CSF neurogranin measurement

The analyses of CSF neurogranin were performed at the Clinical Neurochemistry Laboratory, Sahlgrenska University Hospital, Mölndal, Sweden using an in-house ELISA, based on the NG2 and NG36 antibodies for all samples. Duplicate measures were run with the same batch of reagents. Run acceptance followed accurate criteria and the CV of the duplicate measures were 5.0%. For further details, please see ref. [29].

### Statistical analyses

IBM SPSS version 25 (IBM, Armonk, NY, USA) was used for the statistical analyses.

Parametric tests were applied as the sample size was relatively large and the sample distributions were found to approximate the normal distribution when visually inspected. *T*-tests or one-way ANOVA were employed for the continuous variables, Pearson’s *χ*^2^ for the categorical variables.

Regression analyses were performed with noradrenaline, adrenaline, or dopamine as dependent variables, respectively. In addition to diagnoses, independent variables were included based on biological grounds and comprised Aβ42, P-tau, neurogranin as well as age, gender, and APOEɛ4 genotype.

For analyses including both patients and cognitively unimpaired, Aβ42 and P-tau were dichotomized according to each laboratory’s own cut-offs. P-tau and T-tau were highly correlated both in the patients (*r* = 0.84, *p* < 0.001) and in the cognitively unimpaired (*r* = 0.96, *p* < 0.001). N (T-tau) was therefore not included in the regression analyses as P-tau (T) is regarded more AD specific. Neurogranin is previously shown to correlate with P-tau and T-tau [48]. These markers were also highly correlated in this study (*r* > 0.8, *p* < 0.001 for all groups). Therefore, in the multiple regression analyses only neurogranin was included as a marker of AD related synaptic dysfunction. CSF neurogranin data were not available for 91 memory clinic patients and one cognitively unimpaired. These were excluded from analyses, including CSF neurogranin, but included for other analyses.

The significance level was set at *p* ≤ 0.05.

### Ethical approvals

The study was performed according to the Helsinki declaration. For the patients all participants including the patients’ caregivers gave their consent for participation in writing. All cognitively unimpaired provided written informed consent to participate. The regional Ethics Committee for medical research in the South-East of Norway (REK 2011/2052 and REK 2017/371) and the Data Protector Officer at our institution approved the study.

## Results

### CSF catecholamines are altered in clinical Alzheimer’s disease

The CSF catecholamine concentrations were analyzed in AD patients at the MCI (*n* = 54) and dementia stage (*n* = 240) and cognitively unimpaired (*n* = 113). The cognitively unimpaired were slightly older (*p* = 0.007) and were more educated (*p* < 0.001) than the AD patients. The cohorts are further described in Table 1. CSF noradrenaline and adrenaline correlated among all samples (*r* = 0.55, *p* < 0.001). The correlation was similar within the AD patients (*r* = 0.56, *p* < 0.001 *n* = 294) and cognitively unimpaired (0.57, *p* < 0.001, *n* = 113). CSF dopamine concentrations did not correlate with neither CSF noradrenaline nor CSF adrenaline (for all samples *r* = 0.04, *p* = 0.37 and *r* = 0.02, *p* = 0.65, respectively, *n* = 407).

**Fig. 1:**
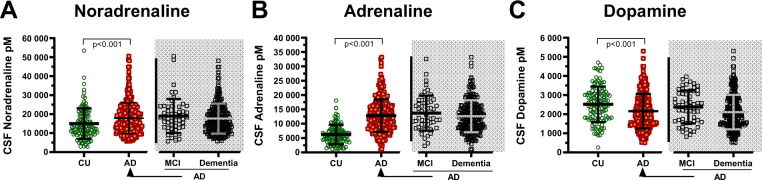
CSF catecholamines and AD clinical presentation. CSF catecholamines in patients with a clinical Alzheimer’s disease presentation. Clinical AD patients at the MCI and dementia stage had similar concentrations of **A** gray area CSF noradrenaline, **B** gray area adrenaline, and **C** gray area dopamine (all *p* > 0.05). These were therefore analyzed as one AD group shown in red color. **A** All AD patients had higher concentrations of noradrenaline compared to the CU. **B** Likewise, all AD patients had higher concentrations of CSF adrenaline than the CU. In contrast, **C** CSF dopamine concentrations were lower among all AD patients than the CU. The larger and smaller lines represent mean and standard deviation respectively, while *p*-values were obtained by *t*-test. AD Alzheimer’s disease, CSF cerebrospinal fluid, CU cognitively unimpaired, MCI mild cognitive impairment, pM picomolar.

Patients in the MCI and dementia stage of AD patients had similar CSF concentrations of noradrenaline, adrenaline, and dopamine (*p* = 0.37, 0.16, and 0.18, respectively, *n* = 240 vs. *n* = 54, Fig. 1 and Table 1). These AD patients were therefore analyzed as one group in the subsequent analyses (*n* = 294). The CSF catecholamine concentrations were altered in symptomatic AD. The AD patients had higher concentrations of noradrenaline and adrenaline in CSF than the cognitively unimpaired (*p* = 0.001 and *p* < 0.001 *n* = 294 vs. *n* = 113). In contrast, the AD patients had lower CSF dopamine concentrations relative to the cognitively unimpaired (*p* < 0.001, *n* = 294 vs. *n* = 113; see Table 1 and Fig. 1).

### CSF catecholamines concentrations in biological Alzheimer’s disease

**Fig. 2:**
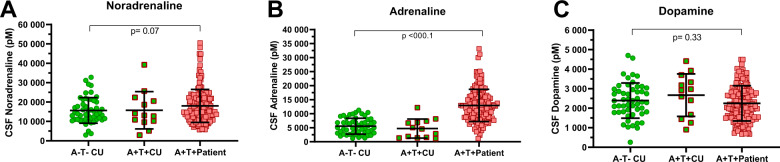
CSF catecholamines in AT refined groups. **A** A+T+ patients did not have statistically significant higher CSF noradrenaline compared to the A−T− cognitively unimpaired (CU). However **B** The CSF adrenaline concentration was higher in A+T+ patients compared to A−T− CU. In contrast **C** CSF dopamine concentrations were similar between A+T+ patients and A−T− CU. **A**–**C** There were no differences in neither CSF noradrenaline, adrenaline nor dopamine between A+T+ and A−T− CU suggesting no alterations prior to symptomatic AD (all *p* > 0.05). The larger and smaller lines represent mean and standard deviation respectively while p-values are obtained by *t*-test. AD Alzheimer’s disease, CSF cerebrospinal fluid, CU cognitively unimpaired.

The clinical presentation is not always reflective of the neuropathology. AD CSF core biomarkers were therefore applied to refine the population into A+T+ AD patients and A+T+ and A−T− cognitively unimpaired, (Table 2). The CSF catecholamine concentrations were unchanged in the AD preclinical phase, as A+T+ and A−T− cognitively unimpaired had similar CSF concentrations of noradrenaline, adrenaline and dopamine (*p* = 0.97 *p* = 0.35, *p* = 0.34 respectively, *n* = 13 vs. *n* = 52, Fig. 2). Reiterating the results of the clinical diagnoses, A+T+ AD patients had higher CSF adrenaline than A−T− cognitively unimpaired (*p* < 0.001 *n* = 151 vs. *n* = 52, Fig. 2). CSF noradrenaline was also higher among A+T+ AD patients than A−T− cognitively unimpaired but did not reach the significance level (*p* = 0.07, Fig. 2). Separate from the two adrenergic transmitters, CSF dopamine concentrations were similar in A+T+ AD patients and A−T− cognitively unimpaired (*p* = 0.33, see Fig. 2).

**Table 2:** Characteristics A−T− cognitively unimpaired and A+T+ patients.

	A+T+ patients	A−T− CU	*p* values
Patient characteristics	*N* = 151	*N* = 52	
Age	70.7 (6.3)	71.3 (5.8)	0.52a
Women	88 (58.3)	22 (42.3)	**0.05b**
Education	12.0 (3.7)	14.7 (3.5)	**<0.001a**
*APOE ε4*-positive*	102 (77.9)	14 (28.0)	**<0.001b**
Cognition			
MMSE	22.6 (4.4)	29.2 (0.9)	**<0.001a**
CDT accepted	72 (48.3)	50 (96.2)	**<0.001b**
TMT A ≥ −2 SD	90 (64.7)	49 (94.2)	**<0.001b**
TMT B ≥ −2 SD	57 (43.5)	48 (92.3)	**<0.001b**
CSF biomarkers			
Noradrenaline pmol/l	18,013.3 (8530.0)	15,682.0 (6604.2)	0.07a
Adrenaline pmol/l	13,213.4 (6588.3)	5571.9 (2815.4)	**<0.001a**
Dopamine pmol/l	2247.8 (902.5)	2390.2 (900.5)	0.33a
Neurogranin pg/ml †	299.6 (74.0)	171.8 (42.2)	**<0.001a**

Bold values identify statistical significance.

Data are presented as *N* (%) and mean (SD).

a = *T*-test, b = Chi square.

*CU* cognitively unimpaired, *MMSE* mini mental state examination, *CDT* clock drawing test, *TMT* trail making test.

^%^Valid percent without missing.

*Missing genotype in 22.

^†^Missing in 53 A+T+ patients.

### Clinical diagnoses and AD related synaptic damage associate with noradrenaline and adrenaline in CSF while dopamine relate to clinical diagnosis but not synaptic damage in adjusted regression analyses

To verify the contribution of clinical diagnosis to the CSF catecholamine levels and to examine other factors, regression analyses were performed with in addition to clinical diagnosis, CSF Aβ42 and P-tau as markers of AD neuropathology and neurogranin as a marker of AD related synaptic damage. These analyses also included age, gender, and *APOEε4* – positivity (Table 3). Due to a high correlation with neurogranin, CSF P-tau was only included in the univariate regressions (see “Methods” section).

**Table 3:** Multiple linear regression.

		Univariate model			Multivariate model	
		*β* (standardized)	*p*	*R*^2^	*β* (standardized)	*p*
Noradrenaline						*R*^2^ = 0.04
Diagnoses (CU = 0, AD = 1)		0.16	**0.001**	0.02	0.19	**0.01**
Age		−0.07	0.19	0.002	−0.03	0.67
Gender (0 = women, 1 = men)		−0.19	**<0.001**	0.03	−0.11	0.08
*APOE* Ɛ4 genotype (0 = neg, 1 = pos)		0.09	0.1	0.005	−0.01	0.84
Amyloid β42 dichotomized		0.06	0.22	0.001	−0.13	0.08
Neurogranin		0.17	**0.002**	0.03	0.14	**0.03**
Phosphorylated tau dichotomized		0.08	0.1	0.004		
Adrenaline						*R*^2^ = 0.31
Diagnoses (CU = 0, AD = 1)		0.48	**<0.001**	0.23	0.54	**<0.001**
Age		−0.13	**0.01**	0.01	−0.09	0.08
Gender (0 = women, 1 = men)		−0.11	**0.03**	0.01	0.03	0.63
*APOE* Ɛ4 genotype (0 = neg, 1 = pos)		0.19	**<0.001**	0.04	0.01	0.92
Amyloid β42 dichotomized		0.23	**<0.001**	0.05	−0.07	0.30
Neurogranin		0.22	**<0.001**	0.04	0.13	**0.01**
Phosphorylated tau dichotomized		0.17	**0.001**	0.03		
Dopamine						*R*^2^ = 0.04
Diagnoses (CU = 0, AD = 1)		−0.17	**<0.001**	0.03	−0.16	**0.03**
Age		0.03	0.51	−0.001	0.004	0.95
Gender (0 = women, 1 = men)		−0.08	0.13	0.003	−0.11	0.08
*APOE* Ɛ4 genotype (0 = neg, 1 = pos)		−0.05	0.39	−0.001	0.02	0.79
Amyloid β1-42 dichotomized		−0.14	**0.005**	0.02	−0.12	0.12
Neurogranin		0.06	0.31	0.00	0.09	0.14
Phosphorylated_1__8__1_ tau dichotomized		0.11	**0.03**	0.009		

Bold values identify statistical significance.

*R*^2^ adjusted R square, *CU* cognitively unimpaired, *AD* Alzheimer’s disease.

Adjusted analyses on the whole sample showed that the two adrenergic transmitters related to synaptic damage and symptomatic AD, as the AD diagnosis and CSF neurogranin both positively associated with CSF noradrenaline and CSF adrenaline (Table 3, *n* = 315). The other co-variates age, gender, *APOEε4*—and CSF Aβ42 positivity were not significant in these analyses. Corresponding analyses of CSF dopamine confirmed the negative association between an AD clinical diagnosis and lower CSF dopamine. Neither CSF neurogranin nor any of the other predictors (age, gender, *APOEε4*—and CSF AΒ42 positivity) were significantly associated with CSF dopamine (Table 3).

To explore if the influence of the co-variates were different dependent on the symptomatic presentation, we performed separate regression analyses of the AD patients and the cognitively unimpaired. Within the AD patients CSF neurogranin positively associated with CSF noradrenaline and CSF adrenaline as also observed in the whole sample. In addition, MCI and lower age were associated with CSF adrenaline. For CSF dopamine, we observed an association with CSF Aβ42 (Supplementary Table 1). Regression analyses within the cognitively unimpaired did not show significance for any of the predictors (data not shown).

### CSF noradrenaline, adrenaline, and dopamine did not relate to behavioral and psychological symptoms in dementia (BPSD)

NPI-Q was included as a measure of BPSD within the AD patients (*n* = 281). As expected, the AD dementia patients had a higher score on the NPI-Q scale than the MCI patients (Table 1). To adjust for a potential influence of BPSD on the results, the NPI-Q score was included in the regression analyses within the AD patients. However, NPI-Q was not a significant predictor for any of the catecholamines, neither in the univariate nor multivariate analyses (Supplementary Table 1).

## Discussion

CSF catecholamines and their metabolites have mainly been studied in solely clinical diagnosed AD patients [32–34]. Recent decades have brought AD research leaps ahead with an increased neuropathological understanding of the disease. This has led to an understanding that AD is not a single clinicobiological entity. We therefore resumed the research question of CSF catecholamine in AD to explore if they relate to the clinical presentation or biological disease. In this study, clinical AD patients had higher CSF noradrenaline and adrenaline concentrations, but lower CSF dopamine concentrations compared to a group of cognitively unimpaired. These associations were upheld in multiple regression analyses adjusting for CSF Aβ42, CSF neurogranin, age, gender, and *APOEε4* positivity. Adjusted analyses showed that besides the AD clinical diagnosis, CSF neurogranin positively associated with CSF noradrenaline and CSF adrenaline but not with CSF dopamine. The positive association between CSF neurogranin and CSF noradrenaline and CSF adrenaline was most prominent in the AD patients and not seen among the cognitively unimpaired alone. NPI-Q was included to rule out that the effect observed of diagnosis truly was an effect relating to BPSD. In AT refined group analyses, only CSF adrenaline was higher in the A+T+ AD patients compared to the A−T− cognitively unimpaired. The result was not significant for CSF noradrenaline in the AT refined analyses that may be due to lower power inferred by the restricted analysis. The refined AT analyses did not show catecholamine alterations in preclinical AD.

Neuronal loss in AD initiates in the preclinical AD stage but is apparent at the prodromal AD stage [14, 15]. This coincides with the alterations in the CSF concentrations of the two adrenergic transmitters. The two adrenergic transmitters correlated in the CSF suggesting simultaneous activity of these neurons. Of the two, adrenaline showed the highest explained variance and related most to AD, in both the clinical and biological perspective. C1 and C2 neurons seems to excite the LC [49, 50] but the functional role of brain adrenaline is elusive [18]. We speculate if the observed higher CSF noradrenaline and adrenaline concentrations in the AD symptomatic phase reflect compensatory mechanisms to AD-induced damage.

Increased responsiveness of the LC in AD [51] may account for higher noradrenaline concentrations. Noradrenaline is widely distributed in the brain through the LCs innervation of several brain areas, including the cortex, hippocampus, and amygdala [5]. These brain areas also show expression of the post-synaptic protein neurogranin [31], which rather specifically increases in AD [52]. The positive association between neurogranin and the two adrenergic transmitters in the CSF points to noradrenaline increasing with AD related synaptic damage. An interpretation may be that signaling failure, due to postsynaptic damage, results in a compensatory increase in CSF noradrenaline release. The LC also suffers early tau pathology and neuronal loss [11, 53], which also may trigger compensatory mechanisms [54, 55]. This aligns with the positive association between CSF P-tau and the two adrenergic transmitters in the univariate regressions and MHPG positively associating with CSF P-tau among the patients [56]. The dynamics of the presynaptic inhibitory receptors apha-2 adrenergic (α2) receptors at Braak NFT stage I-IV are interesting. Compared to middle aged individuals without Aβ-deposits and Braak-stage 0, α2 receptor expression in the hippocampus were higher at Braak NFT stage I while decreased at Braak NFT stage IV [17]. This was observed in asymptomatic individuals indicating that some people tolerate brain pathology better than others, possibly due to a cognitive reserve [57]. These findings are interesting as the upregulation of α2 receptors at earlier Braak NFT stages may be a response to initial increased noradrenaline release in early AD. The decrease at later stages may be a response to reduced noradrenaline release at more progressed AD, as hinted to by biomarker studies [58].

Higher CSF noradrenaline and adrenaline in the AD patients aligns with a recent preliminary study on AD patients of a similar age and at a similar stage as the AD patients here evaluated [35]. However, this result contradicts another study on AD patients at a higher age and at a more severe stage [58]. Neuronal loss and LC shrinkage appear to follow AD progression [16, 59]. As the disease progresses, this may exceed the activated compensatory mechanisms resulting in lower noradrenaline as seen in patients at more advanced stages [60]. Indeed, such dynamics were observed for plasma noradrenaline, AD patients at earlier symptomatic stages had higher concentrations relative to both controls and patients at later stages [61]. We did find in the adjusted analyses, that the MCI patients had higher CSF adrenaline compared to dementia stage AD patients, but this difference was not significant for CSF noradrenaline. Thus, it might be that concentrations of CSF noradrenaline and adrenaline shift in AD, with higher concentrations in the early AD symptomatic phase, which decline as the disease progresses. Contrary to previous findings, we did not find any association between the catecholamines and BPSD [56]. The majority of the patients, however, were in their early phase of the disorder where less BPSD are present, which may explain the lack of an association.

The CSF dopamine concentrations were lower in patients with clinical AD. This aligns with reduced dopamine in AD dementia as reported by brain and CSF concentrations of the dopamine metabolite homovanilic acid (HVA) [62, 63] and supported by a preliminary study by Stefani and colleagues [35]. In contrast to the two adrenergic transmitters, CSF dopamine did not relate to the markers of AD neuropathology, except for Aβ42 within the patient group. Thus, CSF dopamine appears more stably reduced in AD and less related to biological AD. CSF dopamine may rather be associated with cognitive impairment in dementia in general, rather than AD specifically.

LC NFT pathology in asymptomatic individuals is associated with alterations in stress-responses, microglia, and hippocampus tyrosine hydroxylase activity and alterations in α2 adrenergic receptor expression [17]. In young transgenic mice modeling AD neuronal loss in the VTA was followed by astrogliosis [23] suggesting inflammation also in this area as an early event in the pathology. Such alterations may halt but also fuel the disease progress. Here we found no alterations in the CSF concentrations of noradrenaline, adrenaline or dopamine, in preclinical AD as indexed by the CSF AT profile of the cognitively unimpaired. This may indicate that the neurotransmission are later affected. There were however, a limited number of A+T+ cognitively unimpaired and we may have missed effects as we by biomarkers could not stage or fully confirm the neuropathological state. We observed no associations to the neuropathological markers, including CSF neurogranin among all the cognitively unimpaired. Some of these had pathological CSF P-tau concentrations, but as a group, the cognitively unimpaired had non-pathological CSF P-tau concentrations. We speculate on the low levels of CSF P-tau and neurogranin, as appropriate for a control group, not being sufficient to trigger an association to the adrenergic transmitters as seen among the AD patients. The LC appear spared if one ages without AD neuropathology [15] and we did not find an association between the CSF catecholamines and age among the cognitively unimpaired.

A strength of the current study is the fairly large sample size, as the current study included more patients than previous studies. The patients were well defined, as they were recruited from specialized memory clinics and underwent a comprehensive clinical assessment with available biomarkers allowing secondary biomarker refinement. The cognitive unimpaired group went through the same comprehensive assessment.

CSF biomarkers that have shown high accuracy [46, 64] were used to determine the AD neuropathology state. Use of biomarkers provide a strong in vivo indication of the neuropathology but discrepancy between the biomarkers and the actual neuropathology may occur. All patients presented with clinical AD but we cannot rule out that some patients were misdiagnosed, albeit biomarker support, as especially Aβ42 levels may be low even in dementia with Lewy bodies (DLB) [65]. Mixed dementias, such as DLB and AD is common [66]. Cases with DLB pathology might have contributed to the lower dopamine levels in the AD patient group.

Study limitations also include that CSF neurogranin measurements were missing in 91 patients and one cognitive unimpaired. This may have weakened the association found between the CSF catecholamines and neurogranin. That the CSF core biomarkers were analyzed in two different laboratories prevented certain statistical analyses. Furthermore, only cognitively unimpaired and clinical AD patients with and without AT pathology were included in the study. Other pathologies also affect the catecholamine neurons or share traits such as neuroinflammation with AD, but the specificity for AD could here not be assessed. The time of CSF sampling was not matched between the AD patients and the cognitively unimpaired, but all samples were collected during daytime. As this was a cross sectional study including patients with MCI and mostly mild dementia, an evaluation of cognitive changes over time in relation to the catecholamines was not feasible.

## Conclusion

CSF catecholamine concentrations are altered in symptomatic AD from the early phase of the disease. CSF noradrenaline and adrenaline concentrations were higher among AD patients but their temporal dynamics may be non-linear inferring low prognostic and diagnostic value of these transmitters. The two adrenergic transmitters may increase with synaptic damage in symptomatic AD as they positively associated with CSF neurogranin, suggesting similar temporal dynamics but should be confirmed in follow-up studies. This, and the role of the LC in early AD pathology, would be interesting to pursue in future studies. In contrast to CSF noradrenaline and adrenaline, CSF dopamine was lowered in clinical AD and did not relate to the AD neuropathological markers. This indicate CSF dopamine to relate to AD clinical presentation rather than AD neuropathology.

## Supplementary information

Supplementary Table 1.
