# Supplementary material for: Cerebrospinal fluid catecholamines in Alzheimer’s disease patients with and without biological disease
Source: Transl Psychiatry. 2022 Apr 9;12:151. doi: 10.1038/s41398-022-01901-5 (PMC8994756; doi:10.1038/s41398-022-01901-5)
Supplement: Supplementary file 1 — Supplementary Table 1. [file 41398_2022_1901_MOESM1_ESM.docx]

| Supplementary Table 1. Multiple linear regression - the patient cohort | | | | | |  |
| --- | --- | --- | --- | --- | --- | --- |
|  |  | **Univariate** | |  | **Multivariate** | |
|  |  | **standardized β** | **P** | **R square** | **standardized β** | **P** |
| Noradrenaline | |  |  |  |  | R_2_ = 0.04 |
|  | Diagnoses (MCI=1, AD dementia=2) | -0.07 | 0.26 | 0.001 | -0.14 | 0.09 |
|  | Age | -0.08 | 0.18 | 0.003 | -0.11 | 0.16 |
|  | Gender (0=women, 1=men) | -0.22 | **<0.001** | 0.05 | -0.07 | 0.38 |
|  | NPI-Q severity | 0.002 | 0.98 | -0.004 | 0.1 | 0.22 |
|  | *APOE* Ɛ4 genotype (0=neg, 1=pos) | 0.14 | **0.03** | 0.01 | 0.09 | 0.25 |
|  | Amyloid β42 | 0.04 | 0.48 | -0.002 | 0.03 | 0.68 |
|  | Neurogranin | 0.2 | **0.004** | 0.04 | 0.19 | **0.02** |
|  | Phosphorylated tau | 0.18 | **0.002** | 0.03 |  |  |
| Adrenaline |  |  |  |  |  | R_2_ = 0.04 |
|  | Diagnoses (MCI=1, AD dementia=2) | -0.06 | 0.30 | 0.00 | -0.166 | **0.04** |
|  | Age | -0.08 | 0.16 | 0.003 | -0.175 | **0.03** |
|  | Gender (0= women, 1=men) | -0.11 | 0.06 | 0.009 | 0.044 | 0.58 |
|  | NPI-Q severity | -0.043 | 0.47 | 0.002 | -0.009 | 0.91 |
|  | *APOE* Ɛ4 genotype (0=neg, 1=pos) | 0.08 | 0.19 | 0.003 | 0.061 | 0.43 |
|  | Amyloid β42 | 0.01 | 0.82 | -0.003 | 0.031 | 0.71 |
|  | Neurogranin | 0.15 | **0.03** | 0.02 | 0.165 | **0.04** |
|  | Phosphorylated tau | 0.22 | **<0.001** | 0.04 |  |  |
| Dopamine |  |  |  |  |  | R_2_ = 0.04 |
|  | Diagnoses (MCI=1, AD dementia=2) | -0.11 | **0.05** | 0.01 | -0.139 | 0.09 |
|  | Age | 0.05 | 0.43 | -0.001 | 0.054 | 0.50 |
|  | Gender (0=women, 1=men) | -0.06 | 0.33 | 0.00 | -0.07 | 0.37 |
|  | NPI-Q severity | -0.09 | 0.16 | 0.004 | -0.043 | 0.59 |
|  | *APOE* Ɛ4 genotype (0=neg, 1=pos) | -0.06 | 0.35 | 0.00 | -0.072 | 0.35 |
|  | Amyloid β42 | 0.12 | **0.04** | 0.01 | 0.183 | **0.03** |
|  | Neurogranin | 0.09 | 0.19 | 0.004 | 0.132 | 0.1 |
|  | Phosphorylated tau | 0.11 | **0.05** | 0.01 |  |  |

R_2_ = adjusted R square, MCI = cognitively unimpaired, AD = Alzheimer's disease, Amyloid β42 = Amyloid β1-42, Phosphorylated tau = Phosphorylated tau181
